# Clinical Practice Guidelines in Psychiatry: More Confusion Than Clarity? A Critical Review and Recommendation of a Unified Guideline

**DOI:** 10.1155/2014/828917

**Published:** 2014-03-31

**Authors:** Sahoo Saddichha, Santosh K. Chaturvedi

**Affiliations:** ^1^NWMH, Melbourne Health, 4a Devonshire Road, Sunshine, VIC 3020, Australia; ^2^Department of Psychiatry, National Institute of Mental Health & Neurosciences (NIMHANS), Bangalore 560029, India

## Abstract

The discipline of psychiatry has a plethora of guidelines, designed to serve the needs of the clinician. Yet, even a cursory glance is enough to discern the differences between the various guidelines. This paper reviews the current standard guidelines being followed across the world and proposes a unified guideline on the backbone of current evidence and practice being followed. The algorithm for pharmacological and psychosocial treatment for bipolar disorder, major depressive disorder, and schizophrenia is formulated after cross-comparison across four different guidelines and recent meta-analytical evidence. For every disorder, guidelines have different suggestions. Hence, based on the current status of evidence, algorithms have been combined to form a unified guideline for management. Clinical practice guidelines form the basis of standard clinical practice for all disciplines of medicine, including psychiatry. Yet, they are often not read or followed because of poor quality or because of barriers to implementation due to either lack of agreement or ambiguity. A unified guideline can go a long way in helping clear some of the confusion that has crept in due to the use of different guidelines across the world.

## 1. Introduction

Standard clinical practice is considered the hallmark of every clinician, which is usually dictated by adhering to certain clinical guidelines [1]. A good guideline should be able to (a) identify the key decisions (e.g., diagnosis, assessment strategy, and treatment choice), (b) review the relevant, valid evidence on the benefits, risks, and costs of alternative decisions, and (c) present recommendations in a concise, updated format [1, 2]. Guidelines should use best evidence available as well as having the flexibility of being regularly updated, without leaving any scope for ambiguity. They have, therefore, the best potential for ensuring that rigorous clinical standards are maintained and “best practice” is followed by clinicians [2]. Yet, they are often not read or followed because of poor quality [3, 4] or because of barriers to implementation due to either lack of agreement or ambiguity [5]. This issue becomes even more important for trainee residents who are on their way to become consultants as they are required not only to adhere to guidelines while practicing, but also to be fully aware of them during their clinical evaluation.

Psychiatry, as a discipline, is relatively new in the field of evidence based medicine; yet, there exist several clinical guidelines designed to provide guidance of good clinical practice. Some of the most well-known and widely accepted guidelines are the APA guidelines by the American Psychiatric Association [6], the Canadian treatment guidelines by the Canadian Psychiatric Association [7, 8] and the CANMAT (Canadian Network for Mood and Anxiety Treatments) [9, 10], the NICE guidelines by National Institute for Clinical Excellence [11], and the Maudsley guidelines [12]. As should be expected, all the above mentioned guidelines need to be uniform and precise. Yet, as this critique shows, there are several differences when these guidelines are compared. This paper, therefore, attempts to compare the treatment recommendations by the above mentioned guidelines for schizophrenia, major depression, and bipolar disorder, which are often considered as major mental disorders, and evaluates the differences between them. It also, ultimately, suggests a unified guideline putting together the common recommendations and available evidence from all the above.

## 2. Methodology

A Medline search on practice guidelines using terms “schizophrenia,” “depressive disorder,” “bipolar disorder,” “management,” “review,” and combinations thereof helped us retrieve relevant literature. In addition, salient references from obtained articles were followed upon. Standard textbooks of psychiatry (Kaplan & Sadock's Textbook of Psychiatry, Oxford Textbook of Psychiatry and Tasman & Kay's Textbook of Psychiatry) were also screened using the same keywords. The most common guidelines, namely, the CPA, the APA, the CANMAT, NICE, and the Maudsley guidelines, were also reviewed. Additionally, searches were performed in PsycINFO, Medline, Cochrane, and Embase databases for reviewing guidelines for management until June 2011. All literature compiled was then assessed by the authors separately and an algorithm was proposed for each psychiatric disorder, taking into consideration the various guidelines proposed for the management of the specific disorder.

## 3. Results

### 3.1. Management of Bipolar Disorder (Table 1)

#### 3.1.1. Acute Management of Manic Episode

Several differences are apparent in the acute management of a manic episode among the various guidelines. While both the APA and Maudsley prefer mood stabilizers like Lithium or Valproate as first line management, NICE and the Canadian guidelines prefer the use of atypical antipsychotics like Olanzapine (Olanz/OLZ) or Risperidone (Risp). Recent reviews favor the use of either Lithium (Li) or Sodium Valproate (Valp) with/without antipsychotic (Level I evidence) in the management of the acute episode [13, 14]. Since NICE also mentions the utility of both Li and Valp, a unified recommendation is to use Li or Valp as first line and consider Olanz or Risp next.

#### 3.1.2. Acute Management of Mixed Episode

There are no differences of opinion here with all guidelines and hence the unified guideline recommends the use of Valp as a first line management followed by Olanz or Quetiapine (Quet) (Level II evidence [13, 14]).

#### 3.1.3. Acute Management of Depressive Episode

There are minor differences of opinion here, with Li being considered as first line by all the guidelines. However, while the use of Lamotrigine is favored by APA, Maudsley, and the Canadian guidelines, NICE discourages it and prefers a combination of Specific Serotonin Reuptake Inhibitors (SSRIs) with either Li or Valp. The combination of Olanz and Fluoxetine (FXT) has been recommended by almost all guidelines except by NICE, while Quetiapine is universally accepted. Yet, the evidence in favor of these is that of Level II [14, 15]. Therefore the unified guideline recommends the use of Li, either stand-alone or in combination with an SSRI, followed by either Quetiapine or Lamotrigine or the use of Olanz-FXT combination [16].

#### 3.1.4. Acute Management of Rapid Cycling

Only the APA and NICE guidelines comment on this issue with both recommending either Li or Valp or a combination of the two, with Level II evidence available for the same [14]. Additionally, Olanz/Lamotrigine or Quetiapine (Levels II-III evidence [14, 17]) may also be used.

#### 3.1.5. Prophylaxis of Manic Episodes

Lithium and Valp are universally recommended by all the guidelines, which is also the recommendation of the unified guideline.

#### 3.1.6. Prophylaxis of Depressive Episodes

Lamotrigine is recommended as the first line by APA and Canadian guidelines while NICE and Maudsley ask it to be used as second line. Li and Valp have been recommended by all except APA, while Quetiapine has been recommended by both NICE and Maudsley. The unified guideline recommends Lamotrigine followed by Li, Valp, and Quet as drugs to be used in the maintenance of depressive episodes.

#### 3.1.7. Duration of Pharmacotherapy

Only APA and Canadian guidelines have given recommendations on the duration of acute and continuation phase, which, however, vary as seen in the table. The maintenance phase duration, however, is similar across all guidelines and hence the unified guideline recommends treatment in an acute phase up to 12 weeks, in the continuation phase up to 9 months, and in the maintenance phase as 2 years for 1st episode and 5 years to lifetime for subsequent episodes.

#### 3.1.8. Psychosocial Management

The use of psychoeducation has been recommended by only Canadian guidelines with the NICE actively discouraging it. Both NICE and APA as well as recent meta-analysis [14] favor the use of either family focused therapy (FFT) or cognitive behavior therapy (CBT) (Level II evidence), which is the recommendation of the unified guideline also.

### 3.2. Management of Depressive Disorder (Table 2)

#### 3.2.1. Acute Management of Depressive Episode

Since both recent evidence [18–20] and all the guidelines are unanimous in favoring both SSRIs and SNRIs (Serotonin Noradrenaline Reuptake Inhibitors) for the acute management of a depressive episode, this is also the recommendation of the unified guideline.

#### 3.2.2. Management of Atypical Depression

Only the Canadian and NICE guidelines have recommended specific treatments for atypical depression. Recent evidence favors the use of either SSRIs or MAOIs (Monoamine Oxidase Inhibitors) as first line drugs [20], which is also the recommendation of the unified guideline.

#### 3.2.3. Management of Melancholic Depression

Once again, only the Canadian and NICE guidelines have recommended specific treatments for melancholic depression. The unified guideline hence recommends the use of either Paroxetine (PXT) or Venlafaxine (Venla) as first line drugs, while recent reviews have also favored tricyclic antidepressants (TCAs) such as amitriptyline and clomipramine which have both noradrenergic and serotonergic activity [20].

#### 3.2.4. Management of Seasonal Depression

Both the Canadian and NICE recommend the use of either bright light therapy or Fluoxetine (FXT), which is also recommended by the unified guideline.

#### 3.2.5. Management of Anxious Depression

No separate recommendations exist for anxious depression by either APA, NICE, or Maudsley. The Canadian guideline recommends the use of either SSRIs like Paroxetine or Sertraline (SRT) or SNRIs like Venlafaxine. This is also supported by the unified guideline.

#### 3.2.6. Management of Dysthymia

Since no separate recommendations exist except by the Canadian guidelines, the unified guideline recommends the use of either SSRIs or SNRIs as first line management.

#### 3.2.7. Prophylaxis of Depressive Disorder

Almost all the guidelines recommend the continuation of the same antidepressant as was initiated for the acute management of the depressive episode. In addition, the Maudsley guideline recommends the use of either Sertraline, Citalopram, Reboxetine, or Venlafaxine, which have been found effective in prophylaxis. The unified guideline recommends the continuation of the same antidepressant, either SSRI or SNRI.

#### 3.2.8. Duration of Pharmacotherapy

There are differing recommendations on the duration of pharmacotherapy, with both APA and Canadian guidelines specifying the duration of acute phase as up to 12 weeks and continuation phase as up to 9 months. In contrast, the Maudsley and NICE guidelines offer no specific recommendation for the acute management. Long term management again differs, with APA and Canadian being similar and NICE and Maudsley differing on the duration. The unified guideline recommends an acute phase treatment of up to 12 weeks, continuation phase up to 9 months, and maintenance phase as 2 years for a first episode and 5 years to lifetime for subsequent episodes.

#### 3.2.9. Psychosocial Management

CBT and interpersonal therapy (IPT) in 16–20 sessions (Level I evidence) are the recommendation of the APA, Canadian, and the NICE guidelines, as well as that of recent reviews [20] and hence that of the unified guideline.

### 3.3. Management of Schizophrenia (Table 3)

#### 3.3.1. Acute Management of Psychotic Episode

With the exception of the Canadian guidelines, all others recommend the use of either SGAs (first line) or FGAs (second line) as standard drugs. The Canadian guideline only recommends the use of SGAs such as Olanz, Risp, or Quetiapine. Based on recent evidence [21], the unified guideline recommends the use of either first or second generation antipsychotics (SGAs/FGAs) based on clinical and economic needs [22] at a dosage of 300–1000 chlorpromazine (CPZ) equivalents [23].

#### 3.3.2. Prophylaxis of Schizophrenia

All the guidelines and the unified guideline recommend the continued use of the same antipsychotic used to manage the acute episode for prophylaxis [23].

#### 3.3.3. Duration of Pharmacotherapy

The APA and Canadian guidelines recommend similar durations of acute, stabilization, and stable phase treatment. The NICE and Maudsley guidelines recommend acute treatment to last 2 years and give no specific recommendation on duration of prophylaxis. The unified guideline recommends the acute phase treatment to last at least 12 weeks, the stabilization phase to last at least 12 months, and stable phase to last at least 2 years for a first episode and 5 years to lifetime for multiple episodes.

#### 3.3.4. Psychosocial Management

The APA and Canadian guidelines find evidence for and recommend the use of family psychoeducation lasting greater than 9 months, assertive community treatment (ACT), supported employment programs, social skills training (SST), and cognitive behavioral therapy lasting 16–20 sessions. This is also supported by the PORT treatment recommendations [24]. The NICE guideline however finds little or no evidence for psychoeducation and social skills training and hence recommends the use of only 16-session CBT, 10-session family focused therapy, supported employment programs, and Arts therapy. Maudsley guidelines make no specific recommendations. The unified guideline recommendations are therefore the same as the APA guidelines.

## 4. Discussion

Although guidelines have been postulated to improve clinical practice, their implementation has been difficult to achieve due to the characteristics of the guidelines themselves, such as clarity, complexity of treatment recommendations, perceived credibility, use of evidence based medicine, and (pharmaceutical) sponsorship, which have been shown to affect clinicians' acceptance of guidelines [25–28]. To improve acceptance, one would need to formulate an ideal guideline that would be derived from a comprehensive literature review and would explicitly assess the quality of supporting research studies and the methods used for synthesizing evidence. Such a guideline would make recommendations not only for the pharmacological management but also for assessment and psychosocial interventions during both the acute and the maintenance phases of the illness.

Earlier studies have attempted to compare the applicability and evidence strength [29] or the methodological and scientific quality [30] of different guidelines, which have mainly involved schizophrenia. This paper comprehensively looks at and compares the content quality of the four most-followed guidelines for the three most serious mental disorders, namely, schizophrenia, bipolar disorder, and recurrent depressive disorder. It evaluates these guidelines on the presence of evidence based medicine and then makes a unified guideline recommendation, which may be useful to clinicians and students alike. It also observes that most guidelines gave more detailed recommendations in the field of pharmacotherapy while shedding little light on psychosocial management. In contrast to the advice on psychotropic medication, recommendations for psychosocial treatment were very general and nonspecific in many cases. The choice of psychotropic medications was also a major concern. Whereas in some fields recommendations were quite similar among guidelines (e.g., Valproate in case of acute management of mixed episodes, antidepressant use, and duration of long term antipsychotic treatment), others differed widely (recommendations on psychosocial management and duration of acute treatment of mood episode). Such differences actually make it difficult for clinicians to evaluate and implement differing recommendations in their daily practice. The unified guideline attempts to bridge this gap, by evaluation of both the guidelines and available evidence, to make a clear recommendation. The authors hope that this would go a long way in helping clear some of the confusion that has crept in due to the use of different guidelines across the world.

In addition, the authors feel that an internationally acceptable and culturally fair set of recommendations could be developed and form the framework for further elaboration on a national or local basis. This could be facilitated by independent and international organizations such as the WHO and the WPA, after which these could then be used for adaptation to different cultural, economic, and other backgrounds in collaboration with individual stakeholders of the respective countries and regions.

## Figures and Tables

**Table 1:** Comparison of guidelines and unified guideline for the management of bipolar disorder.

	APA	Canadian	NICE	Maudsley	Unified
Bipolar, manic episode-acute treatment	Li/Valp/CBZ ER ± AP (OLZ/Risp/Quet/Arip/Zipr)	OLZ/Risp/Quet	OLZ/Risp/Quet/Li/Valp	Valp/Li/Li or Valp + AP/OLZ/Risp/Quet	Li/Valp/OLZ/Risp/Quet

Bipolar, mixed episode-acute treatment	Valp/Li/CBZ ER ± antipsychotic	Divalproex/OLZ	Valp/OLZ	Valp/OLZ	Valp/OLZ

Bipolar, depressive episode-acute treatment	Li/LMTG/OLZ + FXT/Quet	Li/LMTG/Li + SSRI/Bupropion/OLZ + SSRI/Quet	Li + SSRI/Valp + SSRI/Quet	Li/Li + antidepressant/LMTG/OLZ + FXT/Quet	Li/Li + SSRI/LMTG/OLZ + FXT/Quet

Rapid cycling	Li/Valp/LMTG	No recommendations	Li + Valp	No recommendations	Li/Valp

Maintenance of bipolar, manic episodes	Lithium/OLZ	Li/Divalproex + antipsychotic (Risp/OLZ/Quet)	Lithium/Valp/OLZ	Lithium/Valp/OLZ	Li/Valp/OLZ

Maintenance of bipolar, depressive episodes	LMTG	LMTG/Li/Divalproex + SSRI	Li/Valp + LMTG/Quet	Li/Valp/LMTG/Quet	LMTG/Li/Valp/Quet

Duration	Acute phase: 6 to 12 weeks Continuation phase: 6–9 months Maintenance phase: 1 to 2 years to lifetime	Acute phase: 8 to 12 weeks; continuation phase: 4–6 months Maintenance phase: 1 to 2 years to lifetime	No mention of duration of treatment of acute episode. Long term treatment for at least 2 years after an episode and up to 5 years in case of other risk factors	Same as NICE	Acute phase: up to 12 weeks Continuation phase: up to 9 months. Maintenance phase: 2 years for 1st and 5 years to lifetime for subsequent episodes

Psychosocial management	Family focused therapy	Psychoeducation + CBT/IPT/FFT	FFT/CBT (16–20 sessions)	No recommendations	FFT/CBT

*Li: Lithium; Valp: Sodium Valproate; OLZ: Olanzapine; Risp: Risperidone; CBZ: Carbamazepine; Quet: Quetiapine; Arip: Aripiprazole; Zipr: Ziprasidone; LMTG: Lamotrigine; SSRI: Specific Serotonin Reuptake Inhibitor; FXT: Fluoxetine; CBT: cognitive behavioral therapy; IPT: interpersonal therapy; FFT: family focused therapy.

**Table 2:** Comparison of guidelines and unified guideline for the management of depressive disorder.

	APA	Canadian	NICE	Maudsley	Unified
Depressive episode-acute treatment	SSRI/SNRI/Bupropion/ Mirtazapine	Venlafaxine/SSRIs/novel ADs	SSRIs/SNRIs	SSRIs	SSRIs/SNRIs

Prophylaxis of recurrent depressive disorder	To continue same antidepressant as acute treatment	To continue same antidepressant as acute treatment	To continue same antidepressant as acute treatment	SRT/Citalopram/Reboxetine/ Venlafaxine	Continue SSRIs/SNRIs

Dysthymia	No specific guidelines	SSRIs	No specific guidelines	No specific guidelines	SSRIs/SNRIs

Depression with atypical features	No specific guidelines	FXT/SRT/Moclobemide	SSRIs/Phenelzine	No specific guidelines	SSRIs/MAOIs

Depression with Melancholia	No specific guidelines	PXT/Venlafaxine	PXT/Venla	No specific guidelines	PXT/Venlafaxine

Seasonal depression	No specific guidelines	Bright light therapy/FXT	FXT or bright light therapy	No specific guidelines	FXT/bright light therapy

Anxious depression	No specific guidelines	PXT/SRT/Mirtaz/Venlafaxine	No specific guidelines	No specific guidelines	SSRIs/SNRIs

Duration	Acute phase: 6 to 12 weeks Continuation phase: 6–9 months Maintenance phase: 1 to 2 years to lifetime	Acute phase: 8 to 12 weeks Continuation phase: 4–6 months Maintenance phase: 1 to 2 years to lifetime	No mention of duration of treatment of acute episode Long term treatment for at least 2 years after an episode and up to 5 years in case of other risk factors	Long term treatment for 9 months for first episode and up to 2 years for subsequent episodes	Acute phase: up to 12 weeks Continuation phase: up to 9 months Maintenance phase: 2 years for 1st and 5 years to lifetime for subsequent episodes

Psychosocial management	CBT/IPT	Psychoeducation + CBT/IPT	CBT/IPT (16–20 sessions)	No recommendations	CBT/IPT

*SSRI: Specific Serotonin Reuptake Inhibitor; SNRI: Serotonin Noradrenaline Reuptake Inhibitor; MAOIs: Monoamine Oxidase Inhibitors; SRT: Sertraline; FXT: Fluoxetine; PXT: Paroxetine; CBT: cognitive behavioral therapy; IPT: interpersonal therapy.

**Table 3:** Comparison of guidelines and unified guideline for the management of schizophrenia.

	APA	Canadian	NICE	Maudsley	Unified
Acute treatment of first episode	SGAs/FGAs	Olanz/Risp/Quet	SGAs/FGAs	SGAs/FGAs	SGAs/FGAs

Prophylaxis	To continue same antipsychotic	To continue same antipsychotic	To continue same antipsychotic	To continue same antipsychotic	To continue same antipsychotic

Duration	Acute phase: 4 to 8 weeks Stabilization phase: up to 6 months Stable phase: up to 1 to 1.5 years in first episode, up to 5–10 years in case of 2 or more episodes, and indefinite for multiple prior episodes or more than 2 episodes in 5 years.	Acute phase: 6 to 12 weeks Stabilization phase: 1 year Stable phase: up to 2 years in first episode and up to 5 years in case of multiple episodes.	Acute treatment to last 2 years No duration of long term treatment indicated	Same as NICE	Acute phase: up to 12 weeks Stabilization phase: up to 12 months Stable phase: 2 years for 1st and 5 years to lifetime for subsequent episodes

Psychosocial management	Family psychoeducation (>9 months), assertive community treatment, supported employment, social skills training, and CBT (16–20 sessions)	Supported employment, family psychoeducation, skills training, and CBT	CBT (16 sessions)/FFT (10 sessions)/Arts therapy/supported employment	No recommendations	Family psychoeducation (>9 months), assertive community treatment, supported employment, social skills training, and CBT (16–20 sessions)

SGAs: second generation antipsychotics; FGAs: first generation antipsychotics; OLZ: Olanzapine; Risp: Risperidone; Quet: Quetiapine; CBT: cognitive behavioral therapy; FFT: family focused therapy.

## Clinical Recommendations

- Different guidelines suggest different management strategies for bipolar disorder, major depressive disorder, and schizophrenia.
- A unified guideline, formulated taking into account recent meta-analytical evidence, may help clear the confusion brought about by these differing guidelines.
- International organizations such as the WPA or WHO may help formulate a unified guideline, which may then be modified to meet national or local needs.
